# Diagnostic value of arterial spin labeling for Alzheimer’s disease: A systematic review and meta-analysis

**DOI:** 10.1371/journal.pone.0311016

**Published:** 2024-11-21

**Authors:** Xin-Yue Zhang, Hong Zhang, Qiong-Nan Bao, Zi-Han Yin, Ya-Qin Li, Man-Ze Xia, Zheng-Hong Chen, Wan-Qi Zhong, Ke-Xin Wu, Jin Yao, Fan-Rong Liang

**Affiliations:** 1 School of Acu-Mox and Tuina, Chengdu University of Traditional Chinese Medicine, Chengdu, China; 2 Acupuncture Clinical Research Center of Sichuan Province, Chengdu, China; 3 Traditional Chinese Medicine Hospital of Meishan, Meishan, China; Memorial Sloan Kettering Cancer Center, UNITED STATES OF AMERICA

## Abstract

**Background:**

Arterial spin labeling (ASL) is a magnetic resonance imaging (MRI) technique that offers a non-invasive approach for measuring cerebral blood perfusion (CBF). CBF serves as a marker of neuronal activity, and ASL has demonstrated the potential to detect reductions in CBF associated with early-stage neurodegenerative diseases like Alzheimer’s disease (AD). Consequently, ASL has garnered growing interest as a potential diagnostic tool for AD. Despite the promise of ASL for diagnosing AD, there is a paucity of data regarding the pooled specificity and sensitivity of this technique in this context. The purpose of this systematic review and meta-analysis is to identify the accuracy of ASL in the diagnosis of AD with international clinical diagnosis as the gold standard.

**Methods:**

Four English databases and four Chinese databases were searched from their inception to 30 November 2023. Two independent reviewers extracted relevant information from the eligible articles, while the quality assessment of included studies was assessed using the Quality Assessment of Diagnostic Accuracy Studies 2 (QUADAS-2). The meta-analysis was carried out using the area under the Receiver Operator Characteristic (ROC) curves (AUC) and sensitivity and specificity values. Meta-DiSc 1.4 was used to perform the statistical analysis. STATA 16.0 was used to perform publication bias and sensitivity analysis.

**Results:**

Of 844 relevant articles retrieved, 10 studies involving 494 participants (AD patients = 262, healthy controls = 232) met the inclusion criteria and were included in the meta-analysis. However, the quality of studies was low based on QUADAS-2. The pooled sensitivity, specificity, positive likelihood ratio, negative likelihood ratio, and diagnostic odds ratio of ASL for diagnosing AD was 0.83 (95% CI: 0.78–0.87), 0.81 (95% CI: 0.76–0.86), 4.52 (95% CI: 3.40–6.00), 0.22 (95% CI: 0.17–0.28), and 19.31(95% CI: 12.30–30.31), respectively. The pooled AUC = 0.8932. There was low heterogeneity across the included studies. Finally, sensitivity analysis suggested that the results were reliable.

**Conclusion:**

ASL is an effective and accurate method for the diagnosis of AD. However, due to the limited quantity and quality of the included studies, the above conclusions need to be verified by more studies.

**PROSPERO registration:**

**PROSPERO registration number:** CRD42023484059.

## Introduction

Alzheimer’s disease (AD) is a neurodegenerative disease characterized by progressive cognitive and memory impairment [1], posing a significant public health challenge. In 2021, there were more than 50 million people with AD worldwide [2]. Estimates suggest that by 2050, the number of AD patients in global may reach 10 million [3, 4]. At present, the medical methods for the diagnosis of AD include neuroimaging examination, neuropsychological evaluation, and body fluid markers. Especially in recent years, the rapid development of neuroimaging technology has provided new means for the study of AD. Among them, magnetic resonance imaging (MRI) is more favored by researchers because of its non-radiation and safety characteristics.

Arterial spin labeling (ASL), a MRI technique, can directly assess brain activity by measuring cerebral blood perfusion (CBF). AD is characterized by subtle onset and multifactorial etiology. With the growing recognition of AD’s complexity and multiple causes, many researchers have focused on changes in CBF and its significance in AD’s development [5]. The vascular hypothesis suggested that chronic cerebral hypoperfusion is a key factor in AD [6]. Vascular-derived insults may initiate and/or contribute to neuronal degeneration [7–9]. Epidemiological studies have found that the risk factors of cerebrovascular disease are highly overlapped with AD, such as middle-aged diabetes, hypertension, and obesity [10]. Mouse models implicate capillary disturbances as the precursors to the neurodegenerative changes associated with AD [11, 12].Pathological autopsy also confirmed that there are a variety of pathological changes of vascular diseases in AD, including arterial amyloidosis, capillary degeneration, multiple lacunar infarction [13–15]. Studies [16, 17] have suggested that the changes of CBF may be the initial pathophysiological changes of AD, and the changes of CBF in hippocampus, gray matter and entorhinal cortex may occur before the pathological detection of Aβ and tau protein. ASL potentially detect early signs of nerve degeneration, which has been preliminarily used in the diagnosis of AD patients [18, 19].

In recent years, the research on AD blood perfusion has gradually increased. Such studies pay more attention to single-photon emission computed tomography (SPECT) and positron emission tomography (PET), and ASI is less. However, SPECT and PET have a certain dose of radioactivity, poor tissue resolution, and have an impact on renal function [20]. Therefore, safe, non-invasive, non-radiative, and repeatable ASL technology has attracted attention [21]. Studies have compared the application of ASL with PET and SPECT in the diagnosis of early AD. Takahashi et al. [21] discovered that SPECT and ASL have similar diagnostic capabilities in the diagnosis of AD. Tosun D et al. [22] demonstrated that the diagnostic ability of CBF measured by ASL was comparable to that of brain glucose metabolism measured by FDG-PET. Gaugler et al. [23] demonstrated that various PET approaches, including fluorodeoxyglucose-positron emission tomography (FDG-PET) and SPECT, achieved similar sensitivity (80.0%-100%) and specificity (62.0%-90%) for AD diagnosis. About diagnosing AD, one study reported that [24] an area under the curve (AUC) of 0.94 (95%CI: 0.77, 0.99) for ASL compared to 0.92 (95%CI: 0.76, 0.99) for FDG-PET. However, another study comparing ASL to SPECT reported lower AUCs (0.69 vs. 0.82) [21]. A recent diagnostic comparative meta-analysis of 14 studies [25] demonstrated that FDG-PET still has an advantage over ASL-MRI for diagnosing dementia. As per the summary receiver-operating characteristic (SROC) curve and AUC measures, that FDGPET with an AUC of 86.7%, displays a better diagnostic performance than ASL-MRI with an AUC of 84.2%. The pooled sensitivity of FDG-PET is 0.858, significantly higher than that of ASL-MRI (0.71). Specificity was rather similar among both techniques (0.863 (FDG-PET) versus 0.834 (ASL-MRI)). However, it should be borne in mind that this study included all types of dementia, not just AD. We have doubts about the difference of the above results. The accuracy of ASL in the diagnosis of AD is still unclear. To our knowledge, there is no meta-analysis of the accuracy of ASL in the diagnosis of AD.

Therefore, this systematic review and meta-analysis aim to assess the accuracy of ASL in diagnosing AD, using international clinical diagnosis as the gold standard. This will provide valuable evidence to inform clinical decision-making.

## Methods and analysis

### Design and registration

This study protocol was registered on PROSPERO (registration number: CRD42023484059). We conducted this systematic review and meta-analysis following the guidelines outlined in the Preferred Reporting Items for Systematic Reviews and Meta-Analyses-Diagnostic Test Accuracy (PRISMA-DTA) [26] (S1 File). Ethical approval and informed consent were not required for this study as all data were analyzed from published sources.

### Information sources and search strategy

A computerized search was performed without a limitation on start date for studies published up to November 30, 2023, in the following databases: Chinese Biomedical Literature Database (CBM), China National Knowledge Infrastructure (CNKI), Wanfang Database (WF), VIP Database, Web of Science (WOS), Embase, Cochrane Library, and PubMed. The search was restricted to English and Chinese language publications. The detailed retrieval formula is shown in S2 File.

Two independent reviewers (X-YZ and Q-NB) screened titles, keywords, and abstracts for relevance. Duplicate and ineligible studies were excluded. Full texts of all remaining studies were then reviewed for inclusion. Disagreements were resolved by a third reviewer (Z-HY).

### Inclusion criteria

Studies were selected based on the following criteria: (1) population: patients diagnosed with AD clinical diagnostic criteria, including the NIA-AA [27], NINCDS-ADRDA [28], DSM-IV [29], and DSM-V [30] standards; (2) index test: diagnosis of AD through ASL; (3) comparator test: international clinical diagnostic criteria for AD; and (4) test accuracy or outcome: studies provided the AUC, sensitivity, and specificity data or the corresponding data for a 2 × 2 contingency table construction.

### Exclusion criteria

We excluded studies that met any of the following criteria: (1) conference abstracts, reviews, case reports, editorials, comments, letters, or animal studies; (2) not related to ASL diagnosis of AD; (3) languages other than English or Chinese; or (4) insufficient data to construct a 2 × 2 contingency table.

### Data extraction

Two independent reviewers (X-YZ and Q-NB) used a pre-designed extraction form to collect data on participants, studies, and ASL-MRI imaging features: (1) Participant characteristics: sample size, gender number, average age, years of education; (2) Study characteristics: study origin (first author and country), publication year, study design, AD diagnostic criteria, ASL scan data, sensitivity, specificity, cut-off, AUC, and findings; (3) ASL-MRI imaging features: MRI manufacturers, MRI scan strength (Tesla), ASL sequence, CBF estimation method, and perfusion change. For ASL-MRI imaging features, if a study reported data from multiple brain regions, we prioritized information from the hippocampus. If the hippocampus data was not available, we used the first set of reported information. We also sought missing data or additional information about the study, if needed, from the authors of the included studies via email.

### Quality assessment

Two independent assessors (X-YZ and Q-NB) evaluated the quality of the selected studies using the Quality Assessment of Diagnostic Accuracy Studies 2 (QUADAS-2) tool [31]. The QUADAS-2 tool assesses two key areas: risk of bias and applicability concerns. Risk of bias is evaluated across four domains: patient selection, index test, reference standard, and flow and timing. Similarly, applicability concerns are assessed in three domains: patient selection, index test, and reference standard. Each domain is rated as high risk, low risk, or unclear. Disagreements were resolved by a third reviewer (F-RL or Z-HY). The results were presented using Review Manager 5.3.

### Statistical analysis

Meta-DiSc 1.4 was used to test the heterogeneity, including the heterogeneity caused by threshold effect and non-threshold effect and the source of heterogeneity. Firstly, the summary receiver-operating characteristic (SROC) curve was drawn to determine whether the distribution was ’’shoulder-arm’’, and the Spearman correlation coefficient between the logarithm of sensitivity and the logarithm of (1-specificity) was calculated to analyze the heterogeneity caused by the threshold effect. If the distribution of SROC was shoulder-arm, it suggested that there was a threshold effect; conversely, there was no threshold effect. If there was no threshold effect, the pooled sensitivity, specificity, positive likelihood ratio (PLR), negative likelihood ratio (NLR), and diagnostic odds ratio (DOR) were calculated for the included studies with 95% confidence intervals (CIs), and the AUC. Before the combined analysis, the Cochran’s Q test and I2 test of DOR were calculated to evaluate the heterogeneity caused by non-threshold effect: with the level of statistical significance set at α = 0.05. If *P* > 0.05 and I^2^<50% indicated that the heterogeneity between studies was small, the fixed effect model was used for analysis. On the contrary, it indicated that the heterogeneity was large, and the random effect model was used for analysis. Subgroup analyses and meta-regression analyses were planned to explore potential sources of heterogeneity.

### Publication bias

Deeks’ funnel plot was drawn using STATA 16.0 to assess publication bias [32]. A *p*-value > 0.05 suggested no significant publication bias.

### Sensitivity analysis

Sensitivity analyses were conducted using STATA 16.0. This included two approaches:(1) re-running the analysis after excluding studies with high risk of bias to assess the impact on the overall estimate, and (2) sequentially omitting individual studies to evaluate the influence of any single study on the pooled results.

## Results

### Literature search results

**Fig 1:**
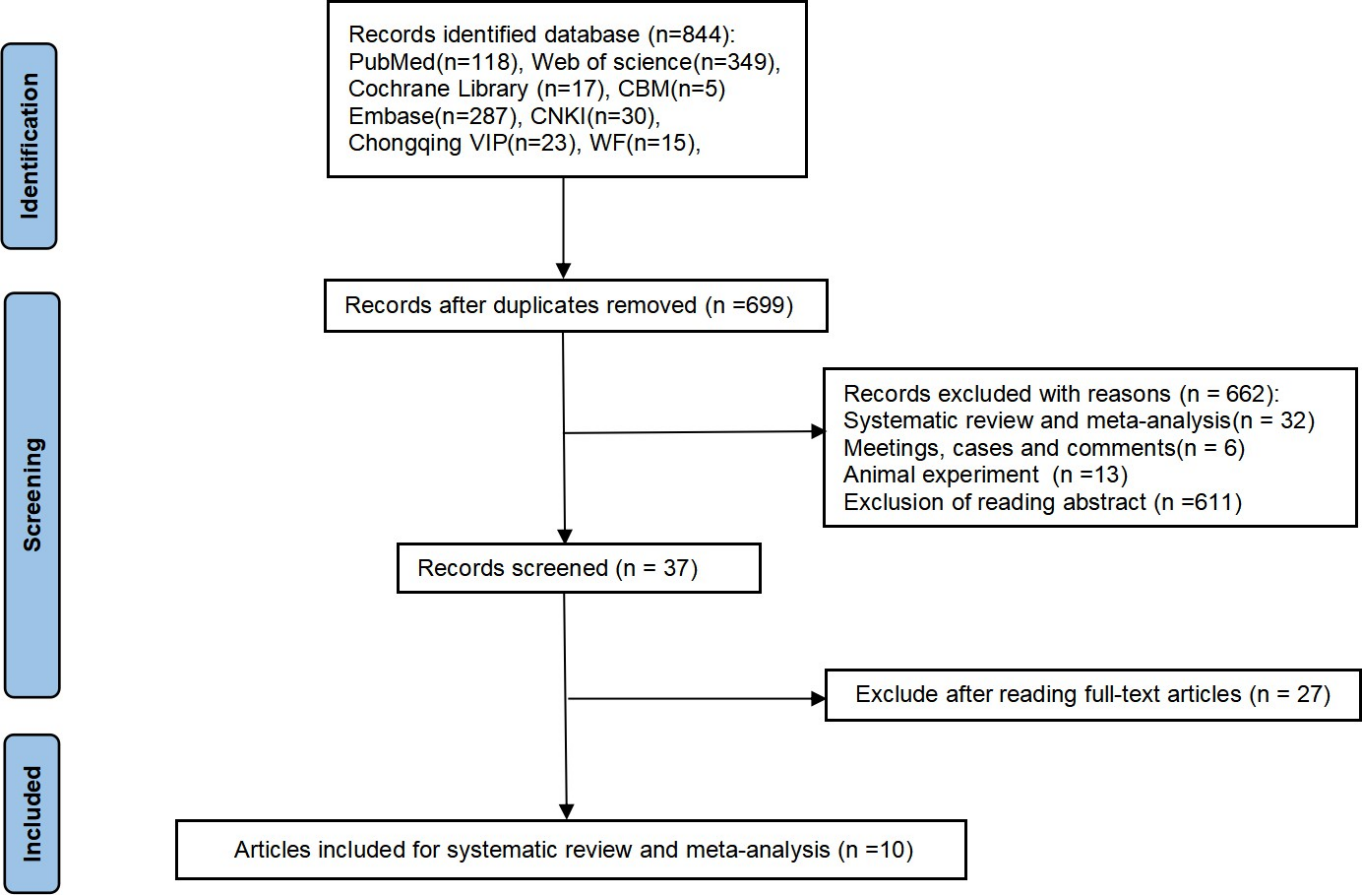
The detailed retrieval process.

Our search identified 844 potentially relevant articles. Applying the pre-defined inclusion criteria, we narrowed the selection to 10 studies involving 494 participants for the final meta-analysis [33–42]. Details of the selection process are available in Fig 1 and S3 File.

### Characteristics of the included studies

Ten studies involving a total of 494 participants (262 patients with AD and 232 healthy controls) were included in the final meta-analysis. Participant characteristics and relevant data are summarized in Tables 1 and 2 and S4 File. Six studies [37–42] were prospective, while the remaining four were retrospective [33–36]. Two studies used NIA-AA [39, 40] as the diagnostic gold standard, 6 studies selected NINCDS-ADRDA [33, 36–38, 41, 42], and two studies selected NINCDS-ADRDA and DSM-IV [34, 35]. Four studies reported cut-off values for the ASL test [36, 38, 39, 41]. MRI scanner manufacturer information was missing for one study [35]. One study used a 1.5 Tesla scanner [33], while all others used 3.0 Tesla scanners. ASL sequences varied across studies: two studies [38, 42] did not report ASL Sequence, one used continuous ASL (CASL) [33], four used pulsed ASL (PASL) [34–37], and three selected pseudocontinuous ASL (pCASL) [39–41]. Five studies [36, 39–42] used the ROIs method to calculate CBF values, three studies used voxel-wise analysis [35, 37, 38], and one study used both methods [34]. One study lacked a clear reporting CBF estimation method [33].

**Table 1:** General characteristics of the included studies.

Author	Country	Diagnostic Criteria	Study Design	AD Group				Normal Control Group				SE (%)	SP (%)	TP	FP	FN	TN	Cut-off	AUC	Extracted Area
				**PP**	**Age (y)**	**Sex (M/F)**	**EY**	**PP**	**Age (y)**	**Sex (M/F)**	**EY**									
Raji CA 2009	USA	NINCDS-ADRDA	RE	13	83.10± 3.35	8/5	/	19	82.31± 3.87	8/11	/	88.00	68.00	11	6	2	13	/	0.790	WB
Yoshiura T 2009	Japan	NINCDS-ADRDA DSM-IV	RE	20	73.50±9.60	10/10	/	23	72.90±6.70	11/12	/	73.90	75.00	15	6	5	17	/	0.861	WB
Dashjamts T 2011	Japan	NINCDS-ADRDA DSM-IV	RE	23	74.65±8.90	9/14	/	23	73.20±6.90	11/12	/	82.60	73.90	19	6	4	17	/	0.893	WB
Mak HK 2014	China	NINCDS-ADRDA	RE	13	/	10/3	/	15	/	14/1	/	92.30	73.30	12	4	1	11	1.396	0.872	LH
Tosun D 2016	USA	NINCDS-ADRDA	PR	28	64.21± 8.17	14/14	16.89 ± 3.22	15	65.80 ± 6.70	9/6	16.8 ± 2.48	86.00	92.00	24	1	4	14	/	0.890	WB
Zheng W 2019	China	NINCDS-ADRDA	PR	40	65.00 ± 10.00	18/12	11.18 ± 3.19	30	64.00 ± 8.00	15/15	12.58 ± 4.60	87.20	86.70	35	4	5	26	0.4723278	0.908	PCC/PCu
Li D 2020	China	NIA-AA	PR	22	71.50±8.40	9/13	/	25	69.30±5.20	10/15	/	81.82	100.0	18	0	4	25	≤40.43	0.930	HP
Sun M 2022	China	NIA-AA	PR	39	68.15 ± 8.71	19/20	10.05 ± 3.53	26	59.77 ± 7.83	9/17	12.35 ± 3.73	92.00	92.00	36	2	3	24	/	0.960	WB
Wang X 2022	China	NINCDS-ADRDA	PR	45	73.51 ± 7.51	16/29	10.82 ± 3.41	33	70.51 ± 7.88	12/21	10.70 ± 3.50	75.60	69.70	34	10	11	23	76.808	0.731	LH
Wang Z 2022	China	NINCDS-ADRDA	PR	19	71.95±7.00	8/11	11.74±3.62	23	66.78±5.87	9/14	11.87±3.06	69.57	84.21	13	4	6	19	/	0.750	LH

**Notes:** RE, Retrospective. PR, Prospective. PP, population. EY, Education Years. SE, Sensitivity. SP, Specificity.WB, whole brain. LH, left hippocampus. HP, hippocampus. PCC, posterior cingulate cortex. PCu, precuneus.

**Table 2:** ASL-MRI characteristics of the included studies.

Author	Manufacturer	Model	MRI Scan Strength (Tesla)	ASL Sequence	CBF Estimation Method	Perfusion Change
Raji CA 2009	GE Discovery	Signa Horizon LX; GE Healthcare, Milwaukee, Wisconsin	1.5 T	CASL	/	/
Yoshiura T 2009	Philips	Achieva Quasar Dual, Philips Medical Systems, Best Netherlands	3.0 T	PASL	voxel-wise,ROIs	SPM analyses revealed focal hypoperfusion in areas over the bilatera precunei and posterior cingulate gyri in AD patients in comparison with control subjects.
Dashjamts T 2011	/	/	3.0 T	PASL	voxel-wise	Significant hypoperfusion, predominantly in the regions of the bilateral precunei and posterior cingulate gyri as well as in the right inferior parietal lobule.
Mak HK 2014	Philips	Achieva, Philips Healthcare, the Netherlands	3.0 T	PASL	ROIs	Right and left hippocampal volumes and middle and posterior cingulate gyri cerebral blood flows were significantly lower in the patients than in the controls.
Tosun D 2016	Siemens	Siemens, Iselin, NJ	3.0 T	PASL	voxel-wise	Compared with controls, AD patients showed special disruptions in rCBF, which were mainly located in the left posterior cingulate cortex, the left and right dorsolateral prefrontal cortex, the left inferior parietal lobule, the right middle temporal gyrus, the left middle occipital gyrus, and the left precuneus.
Zheng W 2019	Siemens	Siemens, Erlangen, Germany	3.0 T	/	voxel-wise	Compared with controls, AD patients showed special disruptions in rCBF, which were mainly located in the left posterior cingulate cortex, the left and right dorsolateral prefrontal cortex, the left inferior parietal lobule, the right middle temporal gyrus, the left middle occipital gyrus, and the left precuneus.
Li D 2020	GE Discovery	GE Discovery MR 750	3.0 T	pCASL	ROIs	Compared with the control group, the CBF of the globus pallidus, putamen, caudate nucleus, hippocampus, thalamus, frontal cortex and parietal cortex in the bilateral brain regions of AD patients were decreased.
Sun M 2022	GE Discovery	SIGNA Premier; GE HealthCare, Milwaukee, WI, United States	3.0 T	pCASL	ROIs	The patients with AD had decreased CBF from 1-delay ASL in all ROIs compared with mild cognitive impairment and normal cognition groups.
Wang X 2022	Siemens	MAGNETOM Prisma, Siemens Healthcare, Erlangen, Germany	3.0 T	pCASL	ROIs	CBF of the right caudate nucleus and left hippocampus was significantly lower in the AD group compared with the normal control group.
Wang Z 2022	GE Discovery	Signa,GE-Healthcare	3.0 T	/	ROIs	The CBF in bilateral posterior cingulate gyrus, left hippocampus and right precuneus was significantly slower in AD group than in control group.

**Notes:** CASL, continuous ASL. pCASL, pseudocontinuous ASL. PASL, pulsed ASL.rCBF, regional cerebral blood flow.

### Quality assessment

**Fig 2:**
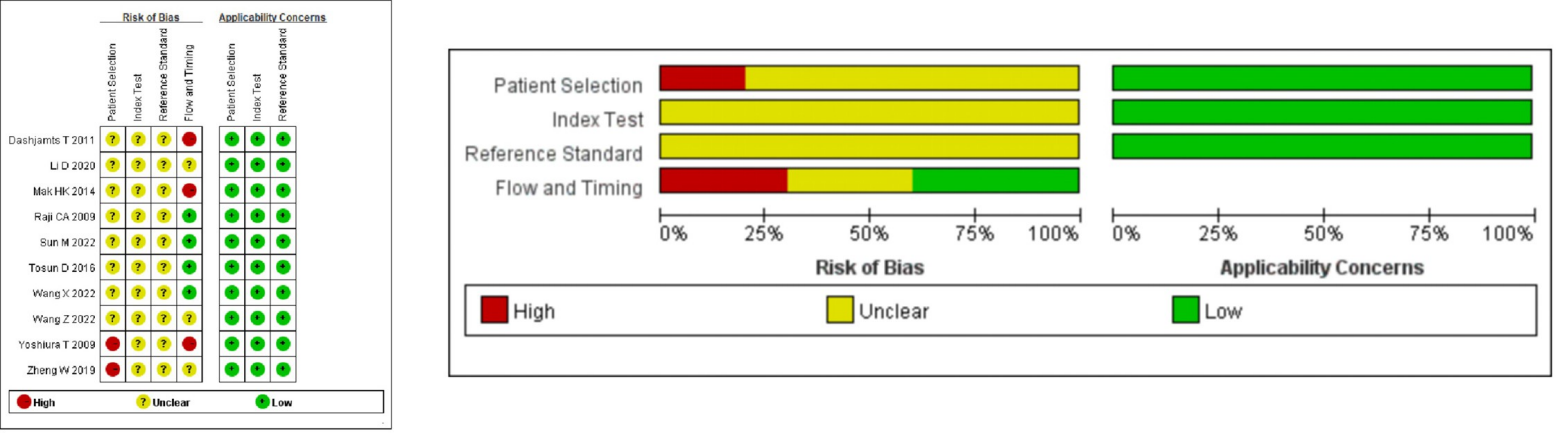
The quality assessment results of the QADAS-2 by review manager 5.3.

The QUADAS-2 assessment results are presented in Fig 2 and S5 File. According to the results, applicability concerns were all low risk. However, in the risk of bias, most studies were unclear, especially in patient selection, index test, and reference standard. Two studies [34, 38] did not avoid the case-control study design, and the remaining studies did not explicitly state this. Additionally, all studies did not indicate whether the interpretation of the results of the index test to be evaluated was performed without knowing the results of the reference standard test, and it was not clear whether blinding was used in the interpretation of the reference standard results. Overall, the quality assessment was not very ideal.

### Data analysis

**Fig 3:**
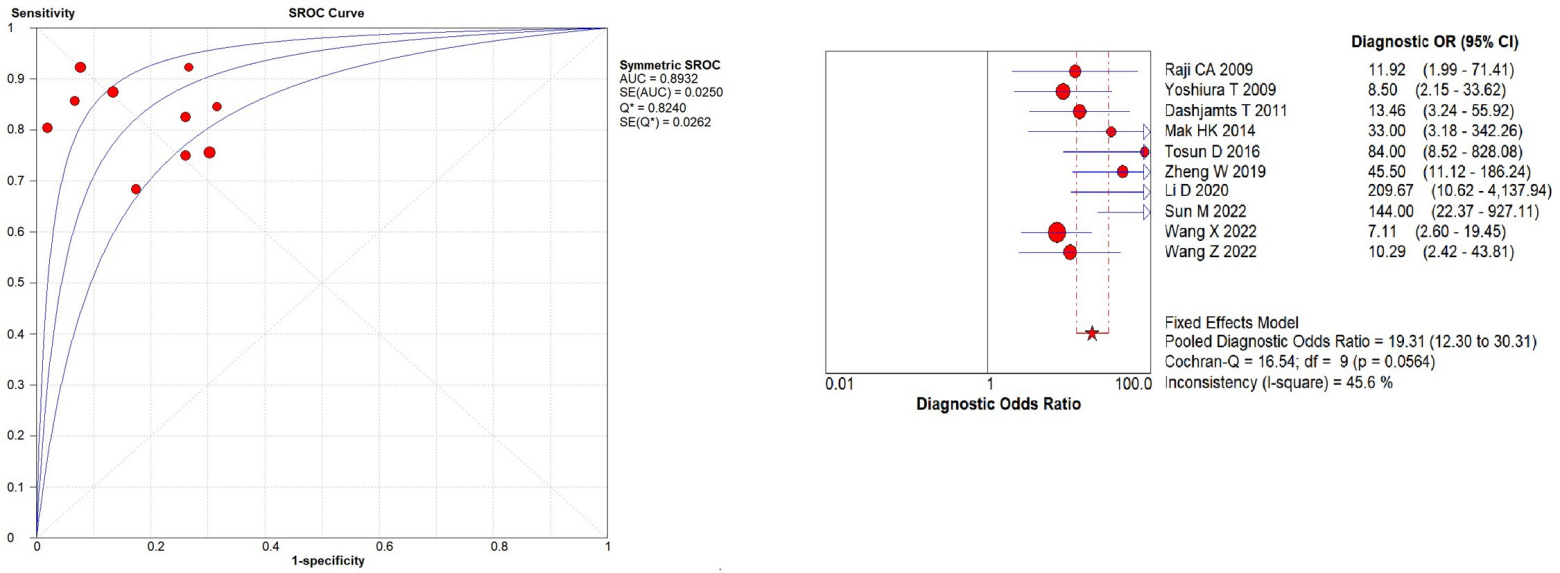
(a). The SROC curve; (b). The diagnostic odds ratio.

**Fig 4:**
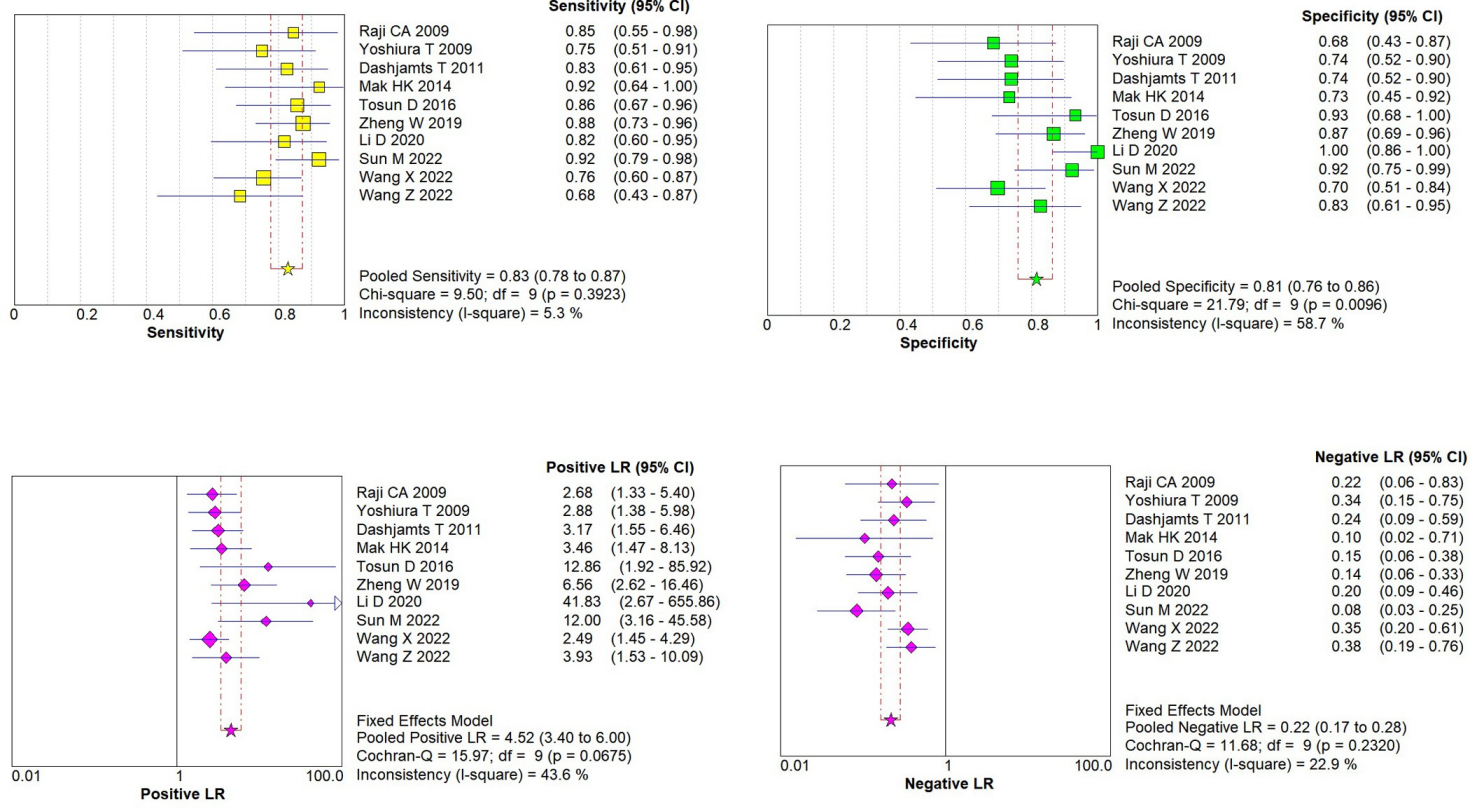
(a). The pooled sensitivity; (b). The pooled specificity; (c). The positive likelihood ratio; (d). The negative likelihood ratio.

Heterogeneity analysis indicated no threshold effect. The SROC curve (Fig 3A) lacked a "shoulder-arm" distribution, and the Spearman correlation coefficient was 0.128 (p = 0.724), supporting the combination of individual studies. Additionally, the Cochran’s Q test for DOR (Fig 3B) showed no significant heterogeneity (Cochran’s Q = 16.54, *p* = 0.0564, I^2^ = 45.6%). Given the low I^2^ value (< 50%), a fixed-effects model was used for the meta-analysis. The pooled estimates using the fixed-effects model revealed promising diagnostic accuracy of ASL for AD: pooled sensitivity = 0.83 (95% CI: 0.78–0.87), pooled specificity = 0.81 (95% CI: 0.76–0.86), PLR = 4.52 (95% CI: 3.40–6.00), NLR = 0.22 (95% CI: 0.17–0.28), and DOR = 19.31 (95% CI: 12.30–30.31). The pooled AUC was 0.8932 with a Q index of 0.8240 (Fig 4A–4D).

### Subgroup analyses and meta-regression

According to the results of data analysis, there was no obvious heterogeneity, and subgroup analysis and meta regression were not needed to explore the source of heterogeneity. However, in order to further explore the influence of grouping factors on the results, we performed subgroup analysis and meta regression with Meta-DiSc 1.4 (Tables 3 and 4). According to the analysis results, the difference in the data extraction area seemed to affect the results. The pooled sensitivity for whole-brain analysis (0.85 [95% CI: 0.78–0.91]) was higher than that for the hippocampus (0.78 [95% CI: 0.68–0.86]). Conversely, the pooled specificity for whole brain (0.80 [95% CI: 0.71–0.87]) was slightly lower than that for the hippocampus (0.81 [95% CI: 0.72–0.88]). In the meta-regression analysis, we assessed the relative diagnostic odds ratio (RDOR) of covariates to identify how a one-unit increase in a covariate might influence the diagnostic performance of the ASL test. After excluding covariates based on *p*-values (starting from the largest *p*-value), the RDOR for the covariate "Extracted Area" was 0.51 (*p* = 0.19), indicating no statistically significant effect.

**Table 3:** Subgroup analyses.

Subgroup	No. of Studies	PL Sensitivity	PL Specificity	PL PLR	PL NLR	PL DOR
Overall	10	0.83 (0.78–0.87)	0.81(0.76–0.86)	4.52(3.40–6.00)	0.22(0.17–0.28)	19.31(12.30–30.31)
Design						
Retrospective	4	0.83 (0.72–0.91)	0.73(0.61–0.82)	3.02(2.08–4.39)	0.24(0.14–0.41)	12.74(5.93–27.37)
Prospective	6	0.83 (0.77–0.88)	0.86(0.80–0.91)	5.78 (3.87–8.63)	0.21(0.15–0.28)	23.87(13.53–42.10)
Country						
China	6	0.83 (0.77–0.88)	0.84 (0.77–0.90)	5.10 (3.52–7.40)	0.21 (0.15–0.29)	22.19(12.56–39.20)
Others	4	0.82 (0.72–0.90)	0.76 (0.65–0.85)	3.65(2.37–5.62)	0.24(0.15–0.36)	14.95 (7.07–31.62)
Standard						
NINCDS-ADRDA	6	0.82 (0.75–0.87)	0.79 (0.71–0.85)	3.91 (2.77–5.51)	0.23 (0.17–0.33)	15.95(9.03–28.18)
Others	4	0.85 (0.76–0.91)	0.86 (0.77–0.92)	5.78(3.51–9.50)	0.19 (0.12–0.30)	26.42(12.46–58.03)
Extracted Area						
Whole brain	5	0.85 (0.78–0.91)	0.80 (0.71–0.87)	4.65 (3.04–7.10)	0.19 (0.12–0.29)	21.65(10.99–42.65)
Hippocampus	4	0.78 (0.68–0.86)	0.81 (0.72–0.88)	3.90 (2.58–5.90)	0.28 (0.19–0.41)	13.94(7.07–27.25)
ASL Sequence						
PASL	4	0.83 (0.74–0.91)	0.78 (0.67–0.86)	3.89 (2.48–6.12)	0.22 (0.13–0.35)	17.51(8.01–38.27)
pCASL	3	0.83 (0.74–0.90)	0.86 (0.76–0.92)	5.36 (3.21–8.94)	0.21 (0.14–0.32)	21.28(9.94–45.55)
CBF Estimation Method						
voxel-wise	3	0.83 (0.78–0.87)	0.81 (0.76–0.86)	4.52 (3.40–6.00)	0.22 (0.17–0.28)	19.31(12.30–30.31)
ROIs	5	0.82 (0.74–0.88)	0.84 (0.76–0.90)	4.80 (3.20–7.19)	0.23 (0.16–0.32)	19.24(10.32–35.89)

**Notes:** PL, Pooled. PLR, positive likelihood ratio. NLR, negative likelihood ratio. DOR, diagnostic odds ratio.

**Table 4:** Meta-regression.

Covariates	Coefficient	Standard Error	P-value	RDOR	[95% CI]
Overall					
Country	-0.183	1.9162	0.9326	0.83	(0.00; 3170.83)
Design	-3.129	3.1247	0.4222	0.04	(0.00; 30208.30)
Standard	-1.663	2.0936	0.5102	0.19	(0.00; 1547.71)
Extracted Area	-2.465	1.5096	0.2442	0.09	(0.00; 56.31)
ASL Sequence	0.950	1.0437	0.4589	2.59	(0.00; 230.59)
CBF Estimation Method	1.725	1.5806	0.3890	5.61	(0.00; 5045.68)
Remove 1					
Design	-3.014	2.5471	0.3219	0.05	(0.00; 162.69)
Standard	-1.593	1.7630	0.4329	0.20	(0.00; 55.59)
Extracted Area	-2.449	1.2343	0.1415	0.09	(0.00; 4.39)
ASL Sequence	0.878	0.8350	0.3701	2.41	(0.00; 34.31)
CBF Estimation Method	1.794	1.0960	0.2002	6.01	(0.00; 196.68)
Remove 2					
Design	-1.082	1.2682	0.4416	0.34	(0.01; 11.46)
Extracted Area	-1.623	0.7368	0.0924	0.20	(0.03; 1.53)
ASL Sequence	0.270	0.4652	0.5932	1.31	(0.36; 4.76)
CBF Estimation Method	1.260	0.8495	0.2121	3.53	(0.33; 37.28)
Remove 3					
Design	-1.066	1.0428	0.3559	0.34	(0.02; 5.10)
Extracted Area	-1.556	0.5858	0.0451	0.21	(0.05; 0.95)
CBF Estimation Method	1.316	0.7115	0.1236	3.73	(0.60; 23.22)
Remove 4					
Extracted Area	-1.252	0.5030	0.0472	0.29	(0.08; 0.98)
CBF Estimation Method	0.748	0.4400	0.1402	2.11	(0.72; 6.20)
Remove 5					
Extracted Area	-0.668	0.4701	0.1984	0.51	(0.17; 1.56)

**Notes:** RDOR, relative diagnostic odds ratio.

### Publication bias

**Fig 5:**
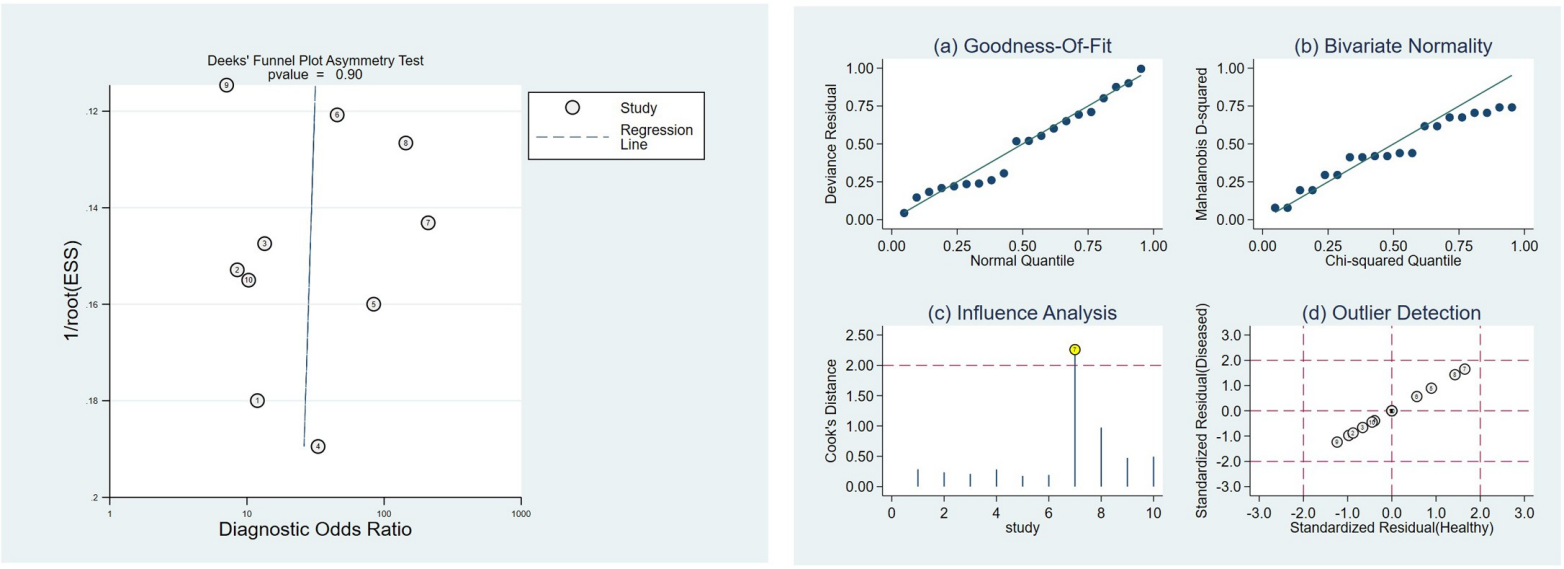
(a). The Deeks’ funnel plot; (b). The sensitivity analysis by STATA.

The Deeks’ funnel plot showed no significant publication bias (*P* = 0.90) (Fig 5A).

## Sensitivity analysis

**Fig 6:**
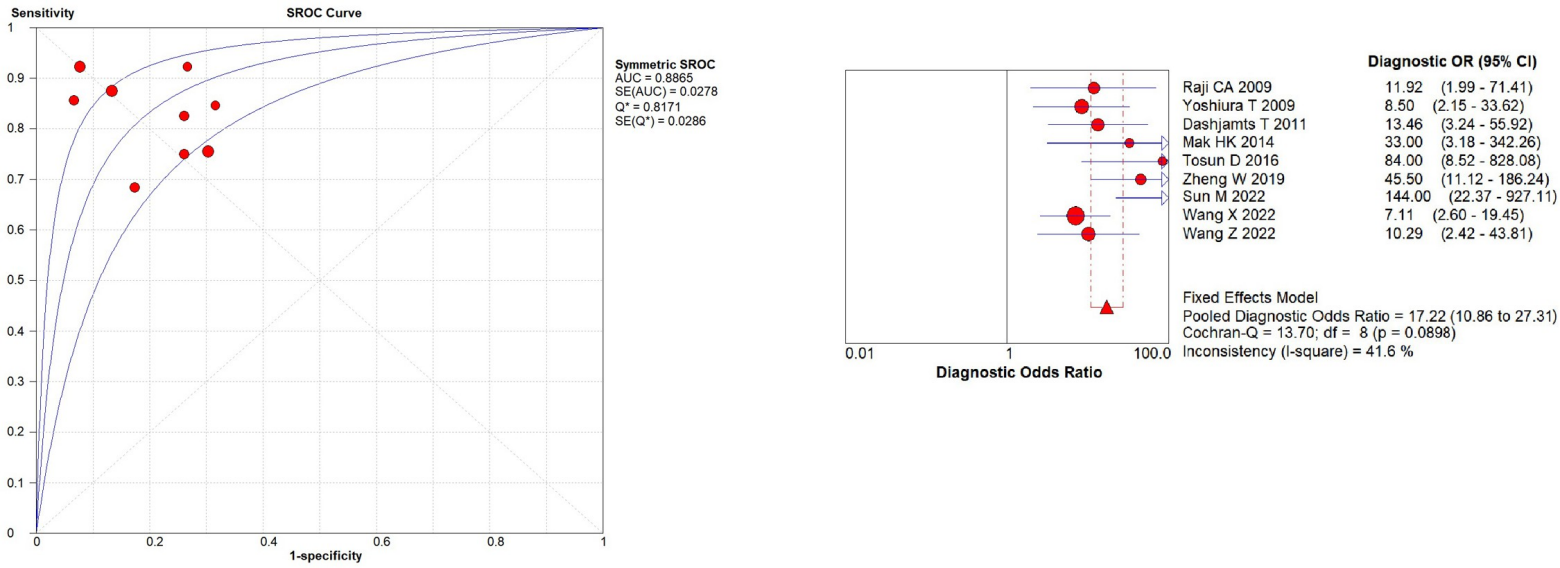
(a). The SROC curve in sensitivity analysis; (b). The diagnostic odds ratio in sensitivity analysis.

Sensitivity analysis with STATA 16.0 revealed a single study with high influence [39] (Fig 5B). After excluding this study, the remaining studies were re-analyzed. The pooled estimates for sensitivity, specificity, PLR, NLR, and DOR of ASL for diagnosing AD remained similar: sensitivity = 0.83 (95% CI: 0.78–0.87), specificity = 0.79 (95% CI: 0.73–0.85), PLR = 4.12 (95% CI: 3.10–5.47), NLR = 0.22 (95% CI: 0.16–0.29), and DOR = 17.22 (95% CI: 10.86–27.31) (Fig 6A and 6B). The pooled AUC was also similar (0.8865) with a Q index of 0.8171. Additionally, sequential omission of individual studies did not significantly alter the overall heterogeneity (Table 5). These findings suggest relatively stable results across the analysis.

**Table 5:** Sensitivity analysis.

Eliminate study	PL Sensitivity	PL Specificity	PL PLR	PL NLR	PL DOR	AUC
Raji CA 2009	0.83 (0.77–0.87)	0.83 (0.77–0.87)	4.75 (3.50–6.43)	4.22 (0.16–0.28)	20.02 (12.53–31.99)	0.8959
Yoshiura T 2009	0.83 (0.78–0.88)	0.82 (0.76–0.87)	4.76 (3.51–6.45)	4.21 (0.15–0.27)	21.44 (13.20–34.82)	0.9026
Dashjamts T 2011	0.83 (0.77–0.87)	0.82 (0.76–0.87)	4.73 (3.48–6.43)	0.22 (0.16–0.29)	20.13 (12.46–32.52)	0.8974
Mak HK 2014	0.82 (0.77–0.87)	0.82 (0.76–0.87)	4.62 (3.43–6.22)	0.22 (0.17–0.29)	18.83 (11.88–29.83)	0.8863
Tosun D 2016	0.82 (0.77–0.87)	0.81 (0.75–0.86)	4.26 (3.21–5.66)	0.22 (0.17–0.30)	17.85 (11.25–28.34)	0.8882
Zheng W 2019	0.82 (0.76–0.87)	0.81 (0.75–0.86)	4.28 (3.18–5.76)	0.23 (0.17–0.31)	17.41 (10.80–28.06)	0.8858
Li D 2020	0.83 (0.78–0.87)	0.79 (0.73–0.85)	4.12 (3.10–5.47)	0.22 (0.16–0.29)	17.22 (10.86–27.31)	0.8865
Sun M 2022	0.81 (0.75–0.86)	0.80 (0.74–0.85)	4.09 (3.07–5.44)	0.24 (0.18–0.32)	16.53 (10.33–26.45)	0.8747
Wang X 2022	0.84 (0.79–0.89)	0.83 (0.78–0.88)	5.24 (3.76–7.29)	0.20 (0.14–0.27)	25.39 (15.00–42.97)	0.9048
Wang Z 2022	0.84 (0.79–0.88)	0.81 (0.75–0.86)	4.57 (3.76–7.30)	0.20 (0.15–0.27)	20.71 (12.76–33.62)	0.9053

**Notes:** PL, Pooled. PLR, positive likelihood ratio. NLR, negative likelihood ratio. DOR, diagnostic odds ratio.

## Discussion

The pathogenesis of AD is complex. Considerable effort over the past 30 years has been targeted at the improved diagnosis of AD [43]. However, current clinical practice relies heavily on clinical diagnosis. This involves identifying patients based on characteristic symptom patterns and excluding other diseases like Parkinson’s disease [44, 45]. Gaugler JE et al. [23] demonstrated significant variability in the sensitivity (53.0%-100%) and specificity (55%-99%) of clinical diagnostic criteria. A major limitation of conventional clinical evaluations is that patients with advanced dementia symptoms may have progressed beyond the point of effective treatment [33].

Neuroimaging techniques offers promising possibility for diagnosing AD before the onset of symptoms or early in the disease course and greatly promotes the research and clinical practice of AD [46, 47]. Structural imaging techniques based on CT and MRI were first applied to the study of AD. They evaluated AD by using structural imaging techniques to quantitatively measure the volume of the hippocampus and medial temporal lobe (MTL). In the current clinical work, the atrophy of hippocampus and MTL has become one of the important neuroimaging indicators for clinical auxiliary diagnosis and differential diagnosis of AD [48]. However, there are some defects in the diagnosis of AD by structural imaging, and the results are easily affected by the clinical experience of clinicians and individual differences of patients [49]. With the development of artificial intelligence (AI) technology, the application of computer-aided diagnosis technology based on medical images in the early diagnosis, disease classification and pathogenesis of AD has received extensive attention [50, 51]. Many widely representative and high-quality medical image data sets have been accumulated. These data sets can continuously train AI programs. For AD, the commonly used MRI datasets have Alzheimer’s disease neuroimaging initiative (ADNI) [52], open access series of imaging studies (OASIS) [53] and minimal interval resonance imaging in Alzheimer’s disease (MIRIAD) [54]. The addition of AI and data sets can better assist doctors in judging the condition. However, the objective identification of biomarkers is one of the important research goals of AD [55]. ASL measures CBF by magnetically labeling arterial water and using it as an endogenous tracer, which can reflect intrinsic functional brain activity at baseline [56]. Among imaging techniques, ASL stands out as a promising functional magnetic resonance imaging (fMRI) method for AD diagnosis due to its non-invasive nature and ability to quantify CBF [57, 58], which is very suitable for screening and longitudinal disease tracking [59].

This study focused solely on the diagnostic value of ASL for AD. Similar to previous studies on the diagnostic accuracy of AD [60, 61], we adopted the international clinical diagnosis of AD as the gold standard. Eickhoff SB et al. [62] suggested that researchers should aim to include at least 20 experiments into an Activation Likelihood Estimation (ALE) meta-analysis to achieve sufficient power for moderate effects. Although our study is not coordinate-based neuroimaging meta-analyses, we can refer to the above conclusions. Only 10 studies were enrolled due to the strict inclusion criteria. Zeng et al. [63] focused on diagnostic accuracy of ASL in detecting the epileptogenic zone which is similar to our study. Only 6 studies were included ultimately, less than 10. The number of studies included in our research is small, and there is research value and significance, but this may affect the universality of the research results. Our systematic review and meta-analysis yielded estimates of pooled sensitivity 0.83 (95% CI, 0.78–0.87), specificity 0.81 (95% CI, 0.76–0.86) (i.e., the missed diagnosis and misdiagnosis rates were 17% and 19%, respectively), DOR 19.31(95% CI: 12.30–30.31), and AUC 0.8932. Compared with the previous diagnostic comparison meta-analysis [25], there was no significant difference in the pooled sensitivity (0.71) and specificity (0.864) of ASL in the diagnosis of dementia. These results indicate that ASL is an effective and accurate method for diagnosing AD. Additionally, Deeks’ funnel plot indicated no significant publication bias. Sensitivity analyses, including excluding highly influential studies and performing one-study-removed analyses, followed by re-analysis, demonstrated no significant heterogeneity. This suggests reliable results. However, the methodological quality of the included studies was not optimal. Despite the absence of initial heterogeneity, we performed subgroup analysis and meta-regression to explore the influence of various factors on the results. Generally, in subgroup analysis, each subgroup has at least 2–3 studies to ensure the reliability of the results. There is also a subgroup analysis of only one study [64]. At least 3 articles in our subgroup analysis are valuable. These analyses, based on study design, country, reference standard, brain region analyzed (extracted area), ASL sequence type, and CBF estimation method, did not reveal any statistically significant differences. Although the above factors have no effect on the results, we found that in the included studies (Table 2), 2 studies [38, 42] did not report ASL sequence. MRI scanner manufacturer information was missing for one study [35]. Another study lacked a clear reporting CBF estimation method [33]. The incompleteness of imaging research information is not conducive to reduction study and meta-analysis. With the development of ASL technology, more imaging studies will be reported in the future. Authoritative imaging reporting standards are urgently needed. This may improve the quality of the research report, which can lay a better foundation for the meta-analysis.

Over the years, several studies have reported ASL findings in patients with clinical AD [18, 57, 58]. Compared to healthy controls, these studies frequently observed reduced blood flow in the posterior cingulate cortex, precuneus, inferior parietal lobe, and lateral prefrontal cortices. However, during data extraction, we noted inconsistencies in the brain regions of interest across studies. In the study of diagnostic accuracy of (18)F amyloid PET tracers for the diagnosis of AD [61], the reference region for data analysis included three different brain regions: cerebellar cortex, whole cerebellum, and occipital corte. Referring to the above research methods, our study combined different brain regions to analyze together. We primarily focused on whole-brain and hippocampal data. The hippocampus is the center of human memory and cognition [65], and is known to be involved in the early stages of AD progression [66]. Rates of whole-brain and hippocampal atrophy are sensitive markers of neurodegeneration [67]. One study solely investigated the posterior cingulate cortex (PCC) and precuneus (Pcu) [38], and we included their data for this specific brain region. According to a recent multimodal meta-analysis [68], Tang X et al. demonstrated that compared with healthy controls, patients with AD showed convergent functional and structural changes in PCC/Pcu and parahippocampal gyrus. This conclusion supports that the brain regions we chose are of analytical significance. Due to the limited number of studies, we were unable to perform a meta-analysis on the diagnostic accuracy of different brain regions. Alsop DC et al. [57] observed the decrease of CBF in multiple brain regions of AD patients through ASL earlier, especially in the precuneus, posterior cingulate gyrus and parietal cortex. Among the nine included studies, all reported differences in cerebral blood flow observed using ASL. Compared to controls, AD patients showed reduced blood flow perfusion in the precuneus [34, 35, 37–38, 42], cingulum gyrus [34–38, 42], inferior parietal lobule [35, 37, 38], lateral prefrontal lobe [37, 38], middle temporal gyrus [37, 38], middle occipital gyrus [37, 38], hippocampus [36, 39, 41, 42], globus pallidus [39], putamen [39], and caudate nucleus [39, 41].

We observed that an emerging trend in the field is the combination of ASL with other modalities for AD diagnosis. For instance, Mak et al. [36] combined ASL with hippocampal volume measured by structural MRI, achieving a combined diagnostic AUC of 0.944. Similarly, Wang et al. [41] demonstrated that a combined model using ASL and T1 mapping improved the ability to distinguish AD from normal controls, with a maximum AUC of 0.94. Dashjamts et al. [35] found that ASL could outperform VBM-based morphological analysis (AUC = 0.893 vs. 0.779) in differentiating AD patients from controls. However, the combination of these two methods (AUC = 0.919) was even more effective. ASL measures CBF which is coupled to neuronal activity. Activation in certain brain regions would normally increases the blood flow to these regions [69]. However, ASL is characterized with low temporal resolution. Therefore, changes with high pace are often not detected or lagged behind [70]. ASL combines other methods to diagnose AD, which can reduce the shortcomings and improve the accuracy. In conclusion, while this study focused solely on the diagnostic value of ASL for AD, the findings suggest that combining ASL with other methods is a promising direction for future research. Further meta-analysis can be conducted on studies of ASL combined with other methods. Secondly, the sensitivity and specificity of ASL in the diagnosis of AD in different brain regions can be studied in the future, but this requires more high-quality basic research to support meta-analysis.

We also acknowledge that our meta-analysis has several limitations. Firstly, the literature search was restricted to published studies. This may have excluded unpublished data and grey literature, potentially introducing publication bias. Secondly, the final number of included studies was limited. Additionally, most studies had small sample sizes, potentially limiting the generalizability of their findings. Furthermore, the methodological quality of the included studies was not optimal, which could affect the reliability of our results. Thirdly, a potential limitation exists in the diagnostic reference standard employed. While all studies utilized international clinical diagnosis of AD as the gold standard, the selection of these criteria varied across the ten included studies. This lack of uniformity weakens the robustness of our analysis as a diagnostic test accuracy. Finally, due to the limited number of studies and variations data processing methods, we did not to explore the diagnostic value of different brain regions.

## Conclusions

Our analysis demonstrates good sensitivity and specificity of ASL for diagnosing AD. However, the generalizability of this conclusion is limited by the number and quality of the included studies. Future research with larger and more robust designs is warranted to confirm these findings. Based on the clinical research of high-quality and standardized report of ASL in the diagnosis of AD in the future, the meta-analysis of ASL combined with other methods for the diagnosis of AD is a potential research direction. Moreover, the pooled sensitivity and specificity of ASL in different brain regions of AD is worth exploring.

## Supporting information

S1 File PRISMA-DTA-checklist. (DOCX)

S2 File Retrieval formula. (DOCX)

S3 File Reasons of excluded studies. (XLS)

S4 File Data extraction EXCEL. (XLSX)

S5 File QUADAS-2 assessment of included studies. (DOCX)
