# Supplementary material for: Diagnostic value of arterial spin labeling for Alzheimer’s disease: A systematic review and meta-analysis
Source: PLoS One. 2024 Nov 21;19(11):e0311016. doi: 10.1371/journal.pone.0311016 (PMC11581220; doi:10.1371/journal.pone.0311016)
Supplement: S2 File — (DOCX) [file pone.0311016.s002.docx]

**Supporting Information 2. Retrieval formula**

**PubMed**

#1 "alzheimer disease"[MeSH Terms]

#2 "alzheimer disease"[Title/Abstract] OR "alzheimer s disease"[Title/Abstract] OR "alzheimer dementia"[Title/Abstract] OR "alzheimer*"[Title/Abstract] OR "alzheimer type dementia"[Title/Abstract] OR "senile dementia"[Title/Abstract]

#3 #1 OR #2

#4 "arterial spin labeling"[Title/Abstract] OR "arterial spin labeling mri"[Title/Abstract] OR "arterial spin labeling"[Title/Abstract] OR "ASL"[Title/Abstract]

#5 "Diagnosis"[Title/Abstract] OR "marker"[Title/Abstract] OR "Subject"[Title/Abstract] OR "Identified"[Title/Abstract] OR "specificity"[Title/Abstract] OR "sensitivity"[Title/Abstract]

#6 #3 AND #4 AND #5

**Web of science**

TS=((‘Alzheimer Disease’ OR ‘Alzheimer's Disease’ OR ‘Alzheimer Dementia’ OR ‘Alzheimer*’ OR ‘Alzheimer Type Dementia’ OR ‘Senile Dementia’) AND (‘arterial spin labeling’ OR ‘arterial spin labeling mri’ OR ‘arterial spin labeling’ OR ‘ASL’ ) AND (‘Diagnosis’ OR ‘marker’ OR ‘Subject’ OR ‘Identified’ OR ‘specificity’ OR ‘sensitivity’ ))

**Cochrane Library**

#1 MeSH descriptor: [Alzheimer Disease] explode all trees

#2 alzheimer disease:ti,ab,kw OR alzheimers disease:ti,ab,kw OR alzheimer dementia:ti,ab,kw OR alzheimer type dementia:ti,ab,kw OR senile dementia

#3 #1 OR #2

#4 arterial spin labeling:ti,ab,kw OR arterial spin labeling mri:ti,ab,kw OR ASL

#5 Diagnosis:ti,ab,kw OR marker:ti,ab,kw OR Subject:ti,ab,kw OR Identified:ti,ab,kw OR specificity:ti,ab,kw OR sensitivity

#6 #3 AND #4 AND #5

**Embase**

#1 'alzheimer disease'/exp OR 'alzheimer disease'

#2 'alzheimer disease':ti,ab,kw OR 'alzheimers disease':ti,ab,kw OR 'alzheimer dementia':ti,ab,kw OR 'alzheimer type dementia':ti,ab,kw OR 'senile dementia':ti,ab,kw

#3 #1 OR #2

#4 'arterial spin labeling':ti,ab,kw OR 'arterial spin labeling mri':ti,ab,kw OR 'ASL'

#5 'Diagnosis':ti,ab,kw OR 'marker':ti,ab,kw OR 'Subject':ti,ab,kw OR 'Identified':ti,ab,kw OR 'specificity':ti,ab,kw OR 'sensitivity'

#6 #3 AND #4 AND #5

**CNKI**

(SU='动脉自选标记' + '动脉自旋标记成像' + '动脉自旋标记灌注' + 'ASL') AND SU=('阿尔茨海默' + '老年痴呆' + '阿尔兹海默' + '老年性痴呆') AND SU= ('诊断' + '标志物' + '标记物' + '明确' + '灵敏度' + '特异度')

**WF**

题名或关键词:(阿尔茨海默 OR 阿尔兹海默 OR 老年痴呆 OR 老年性痴呆) and 题名或关键词:(动脉自选标记 OR 动脉自旋标记成像 OR 动脉自旋标记灌注 OR ASL) and 题名或关键词:(诊断 OR 标志物 OR 标记物 OR 灵敏度 OR 特异度)

**Chongqing VIP**

M= (阿尔茨海默 OR 阿尔兹海默 OR 老年痴呆 OR 老年性痴呆) and M= (动脉自选标记 OR 动脉自旋标记成像 OR 动脉自旋标记灌注 OR ASL) and M= (诊断 OR 标志物 OR 标记物 OR 灵敏度 OR 特异度)

**CBM**

( "阿尔茨海默"[标题:智能] OR "阿尔兹海默"[标题:智能] OR "老年痴呆"[标题:智能] OR "老年性痴呆"[标题:智能]) AND( "动脉自选标记"[标题:智能] OR "动脉自旋标记成像"[标题:智能] OR "动脉自旋标记灌注"[标题:智能] OR " ASL"[标题:智能]) AND( "诊断"[摘要:智能] OR "标志物"[摘要:智能] OR "标记物"[摘要:智能] OR "灵敏度"[摘要:智能] OR "特异度"[摘要:智能])
