# Supplementary material for: Diagnostic value of arterial spin labeling for Alzheimer’s disease: A systematic review and meta-analysis
Source: PLoS One. 2024 Nov 21;19(11):e0311016. doi: 10.1371/journal.pone.0311016 (PMC11581220; doi:10.1371/journal.pone.0311016)
Supplement: S5 File — (DOCX) [file pone.0311016.s005.docx]

**Supporting Information 5: QUADAS-2 assessment of included studies.**

1. **Raji CA 2009:**

**Risk of bias:**

| **Patient selection** | Was a consecutive or random sample of patients enrolled? | No | Unclear |
| --- | --- | --- | --- |
|  | Was a case-control design avoided? | Unclear |  |
|  | Did the study avoid inappropriate exclusions? | No |  |
| **Index test** | Were the index test results interpreted without knowledge of the results of the reference standard? | Unclear | Unclear |
|  | If a threshold was used, was it pre-specified? | Unclear |  |
| **Reference standard** | Is the reference standard likely to correctly classify the target condition? | Yes | Unclear |
|  | Were the reference standard results interpreted without knowledge of the results of the index test? | Unclear |  |
| **Flow and timing** | Was there an appropriate interval between index test(s) and reference standard? | Yes | Low risk |
|  | Did all patients receive a reference standard? | Yes |  |
|  | Did all patients receive the same reference standard? | Yes |  |
|  | Were all patients included in the analysis? | Yes |  |

**Applicability concerns:**

| **Patient selection** | Are there concerns that the included patients do not match the review question? | No | Low risk |
| --- | --- | --- | --- |
| **Index test** | Are there concerns that the index test, its conduct, or interpretation differ from the review question? | No | Low risk |
| **Reference standard** | Are there concerns that the target condition as defined by the reference standard does not match the review question? | No | Low risk |

**2. Yoshiura T 2009:**

**Risk of bias:**

| **Patient selection** | Was a consecutive or random sample of patients enrolled? | No | High risk |
| --- | --- | --- | --- |
|  | Was a case-control design avoided? | Unclear |  |
|  | Did the study avoid inappropriate exclusions? | No |  |
| **Index test** | Were the index test results interpreted without knowledge of the results of the reference standard? | Unclear | Unclear |
|  | If a threshold was used, was it pre-specified? | Unclear |  |
| **Reference standard** | Is the reference standard likely to correctly classify the target condition? | Yes | Unclear |
|  | Were the reference standard results interpreted without knowledge of the results of the index test? | Unclear |  |
| **Flow and timing** | Was there an appropriate interval between index test(s) and reference standard? | Unclear | High risk |
|  | Did all patients receive a reference standard? | No |  |
|  | Did all patients receive the same reference standard? | No |  |
|  | Were all patients included in the analysis? | No |  |

**Applicability concerns:**

| **Patient selection** | Are there concerns that the included patients do not match the review question? | No | Low risk |
| --- | --- | --- | --- |
| **Index test** | Are there concerns that the index test, its conduct, or interpretation differ from the review question? | No | Low risk |
| **Reference standard** | Are there concerns that the target condition as defined by the reference standard does not match the review question? | No | Low risk |

**3. Dashjamts T 2011:**

**Risk of bias:**

| **Patient selection** | Was a consecutive or random sample of patients enrolled? | No | Unclear |
| --- | --- | --- | --- |
|  | Was a case-control design avoided? | Yes |  |
|  | Did the study avoid inappropriate exclusions? | Yes |  |
| **Index test** | Were the index test results interpreted without knowledge of the results of the reference standard? | Unclear | Unclear |
|  | If a threshold was used, was it pre-specified? | Unclear |  |
| **Reference standard** | Is the reference standard likely to correctly classify the target condition? | Yes | Unclear |
|  | Were the reference standard results interpreted without knowledge of the results of the index test? | Unclear |  |
| **Flow and timing** | Was there an appropriate interval between index test(s) and reference standard? | Unclear | High risk |
|  | Did all patients receive a reference standard? | No |  |
|  | Did all patients receive the same reference standard? | No |  |
|  | Were all patients included in the analysis? | No |  |

**Applicability concerns:**

| **Patient selection** | Are there concerns that the included patients do not match the review question? | No | Low risk |
| --- | --- | --- | --- |
| **Index test** | Are there concerns that the index test, its conduct, or interpretation differ from the review question? | No | Low risk |
| **Reference standard** | Are there concerns that the target condition as defined by the reference standard does not match the review question? | No | Low risk |

**4. Mak HK 2014:**

**Risk of bias:**

| **Patient selection** | Was a consecutive or random sample of patients enrolled? | No | Unclear |
| --- | --- | --- | --- |
|  | Was a case-control design avoided? | Yes |  |
|  | Did the study avoid inappropriate exclusions? | Yes |  |
| **Index test** | Were the index test results interpreted without knowledge of the results of the reference standard? | Unclear | Unclear |
|  | If a threshold was used, was it pre-specified? | No |  |
| **Reference standard** | Is the reference standard likely to correctly classify the target condition? | Yes | Unclear |
|  | Were the reference standard results interpreted without knowledge of the results of the index test? | Unclear |  |
| **Flow and timing** | Was there an appropriate interval between index test(s) and reference standard? | Unclear | High risk |
|  | Did all patients receive a reference standard? | Yes |  |
|  | Did all patients receive the same reference standard? | Yes |  |
|  | Were all patients included in the analysis? | No |  |

**Applicability concerns:**

| **Patient selection** | Are there concerns that the included patients do not match the review question? | No | Low risk |
| --- | --- | --- | --- |
| **Index test** | Are there concerns that the index test, its conduct, or interpretation differ from the review question? | No | Low risk |
| **Reference standard** | Are there concerns that the target condition as defined by the reference standard does not match the review question? | No | Low risk |

**5. Tosun D 2016:**

**Risk of bias:**

| **Patient selection** | Was a consecutive or random sample of patients enrolled? | Unclear | Unclear |
| --- | --- | --- | --- |
|  | Was a case-control design avoided? | Unclear |  |
|  | Did the study avoid inappropriate exclusions? | No |  |
| **Index test** | Were the index test results interpreted without knowledge of the results of the reference standard? | Unclear | Unclear |
|  | If a threshold was used, was it pre-specified? | Unclear |  |
| **Reference standard** | Is the reference standard likely to correctly classify the target condition? | Yes | Unclear |
|  | Were the reference standard results interpreted without knowledge of the results of the index test? | Unclear |  |
| **Flow and timing** | Was there an appropriate interval between index test(s) and reference standard? | Yes | Low risk |
|  | Did all patients receive a reference standard? | Yes |  |
|  | Did all patients receive the same reference standard? | Yes |  |
|  | Were all patients included in the analysis? | Yes |  |

**Applicability concerns:**

| **Patient selection** | Are there concerns that the included patients do not match the review question? | No | Low risk |
| --- | --- | --- | --- |
| **Index test** | Are there concerns that the index test, its conduct, or interpretation differ from the review question? | No | Low risk |
| **Reference standard** | Are there concerns that the target condition as defined by the reference standard does not match the review question? | No | Low risk |

**6. Zheng W 2019:**

**Risk of bias:**

| **Patient selection** | Was a consecutive or random sample of patients enrolled? | No | High risk |
| --- | --- | --- | --- |
|  | Was a case-control design avoided? | Unclear |  |
|  | Did the study avoid inappropriate exclusions? | No |  |
| **Index test** | Were the index test results interpreted without knowledge of the results of the reference standard? | Unclear | Unclear |
|  | If a threshold was used, was it pre-specified? | Unclear |  |
| **Reference standard** | Is the reference standard likely to correctly classify the target condition? | Yes | Unclear |
|  | Were the reference standard results interpreted without knowledge of the results of the index test? | Unclear |  |
| **Flow and timing** | Was there an appropriate interval between index test(s) and reference standard? | Unclear | Unclear |
|  | Did all patients receive a reference standard? | Yes |  |
|  | Did all patients receive the same reference standard? | Yes |  |
|  | Were all patients included in the analysis? | Yes |  |

**Applicability concerns:**

| **Patient selection** | Are there concerns that the included patients do not match the review question? | No | Low risk |
| --- | --- | --- | --- |
| **Index test** | Are there concerns that the index test, its conduct, or interpretation differ from the review question? | No | Low risk |
| **Reference standard** | Are there concerns that the target condition as defined by the reference standard does not match the review question? | No | Low risk |

**7. Li D 2020:**

**Risk of bias:**

| **Patient selection** | Was a consecutive or random sample of patients enrolled? | Unclear | Unclear |
| --- | --- | --- | --- |
|  | Was a case-control design avoided? | Unclear |  |
|  | Did the study avoid inappropriate exclusions? | No |  |
| **Index test** | Were the index test results interpreted without knowledge of the results of the reference standard? | Unclear | Unclear |
|  | If a threshold was used, was it pre-specified? | Unclear |  |
| **Reference standard** | Is the reference standard likely to correctly classify the target condition? | Yes | Unclear |
|  | Were the reference standard results interpreted without knowledge of the results of the index test? | Unclear |  |
| **Flow and timing** | Was there an appropriate interval between index test(s) and reference standard? | Unclear | Unclear |
|  | Did all patients receive a reference standard? | Yes |  |
|  | Did all patients receive the same reference standard? | Yes |  |
|  | Were all patients included in the analysis? | Yes |  |

**Applicability concerns:**

| **Patient selection** | Are there concerns that the included patients do not match the review question? | No | Low risk |
| --- | --- | --- | --- |
| **Index test** | Are there concerns that the index test, its conduct, or interpretation differ from the review question? | No | Low risk |
| **Reference standard** | Are there concerns that the target condition as defined by the reference standard does not match the review question? | No | Low risk |

**8. Sun M 2022:**

**Risk of bias:**

| **Patient selection** | Was a consecutive or random sample of patients enrolled? | Unclear | Unclear |
| --- | --- | --- | --- |
|  | Was a case-control design avoided? | Unclear |  |
|  | Did the study avoid inappropriate exclusions? | No |  |
| **Index test** | Were the index test results interpreted without knowledge of the results of the reference standard? | Unclear | Unclear |
|  | If a threshold was used, was it pre-specified? | Unclear |  |
| **Reference standard** | Is the reference standard likely to correctly classify the target condition? | Yes | Unclear |
|  | Were the reference standard results interpreted without knowledge of the results of the index test? | Unclear |  |
| **Flow and timing** | Was there an appropriate interval between index test(s) and reference standard? | Yes | Low risk |
|  | Did all patients receive a reference standard? | Yes |  |
|  | Did all patients receive the same reference standard? | Yes |  |
|  | Were all patients included in the analysis? | Yes |  |

**Applicability concerns:**

| **Patient selection** | Are there concerns that the included patients do not match the review question? | No | Low risk |
| --- | --- | --- | --- |
| **Index test** | Are there concerns that the index test, its conduct, or interpretation differ from the review question? | No | Low risk |
| **Reference standard** | Are there concerns that the target condition as defined by the reference standard does not match the review question? | No | Low risk |

**9. Wang X 2022:**

**Risk of bias:**

| **Patient selection** | Was a consecutive or random sample of patients enrolled? | Unclear | Unclear |
| --- | --- | --- | --- |
|  | Was a case-control design avoided? | Unclear |  |
|  | Did the study avoid inappropriate exclusions? | No |  |
| **Index test** | Were the index test results interpreted without knowledge of the results of the reference standard? | No | Unclear |
|  | If a threshold was used, was it pre-specified? | Unclear |  |
| **Reference standard** | Is the reference standard likely to correctly classify the target condition? | Yes | Unclear |
|  | Were the reference standard results interpreted without knowledge of the results of the index test? | No |  |
| **Flow and timing** | Was there an appropriate interval between index test(s) and reference standard? | Yes | Low risk |
|  | Did all patients receive a reference standard? | Yes |  |
|  | Did all patients receive the same reference standard? | Yes |  |
|  | Were all patients included in the analysis? | Yes |  |

**Applicability concerns:**

| **Patient selection** | Are there concerns that the included patients do not match the review question? | No | Low risk |
| --- | --- | --- | --- |
| **Index test** | Are there concerns that the index test, its conduct, or interpretation differ from the review question? | No | Low risk |
| **Reference standard** | Are there concerns that the target condition as defined by the reference standard does not match the review question? | No | Low risk |

**10. Wang Z 2022:**

**Risk of bias:**

| **Patient selection** | Was a consecutive or random sample of patients enrolled? | Unclear | Unclear |
| --- | --- | --- | --- |
|  | Was a case-control design avoided? | Unclear |  |
|  | Did the study avoid inappropriate exclusions? | No |  |
| **Index test** | Were the index test results interpreted without knowledge of the results of the reference standard? | Unclear | Unclear |
|  | If a threshold was used, was it pre-specified? | Unclear |  |
| **Reference standard** | Is the reference standard likely to correctly classify the target condition? | Yes | Unclear |
|  | Were the reference standard results interpreted without knowledge of the results of the index test? | Unclear |  |
| **Flow and timing** | Was there an appropriate interval between index test(s) and reference standard? | Unclear | Unclear |
|  | Did all patients receive a reference standard? | Yes |  |
|  | Did all patients receive the same reference standard? | Yes |  |
|  | Were all patients included in the analysis? | Yes |  |

**Applicability concerns:**

| **Patient selection** | Are there concerns that the included patients do not match the review question? | No | Low risk |
| --- | --- | --- | --- |
| **Index test** | Are there concerns that the index test, its conduct, or interpretation differ from the review question? | No | Low risk |
| **Reference standard** | Are there concerns that the target condition as defined by the reference standard does not match the review question? | No | Low risk |
